# Molecular landscape and targeted therapy of acute myeloid leukemia

**DOI:** 10.1186/s40364-018-0146-7

**Published:** 2018-11-08

**Authors:** Runxia Gu, Xue Yang, Hui Wei

**Affiliations:** Leukemia Center, Institute of Hematology and Blood Diseases Hospital, Chinese Academy of Medical Sciences & Peking Union Medical College, Tianjin, 300020 People’s Republic of China

**Keywords:** Acute myeloid leukemia, Molecular landscape, Targeted therapy

## Abstract

For decades, genetic aberrations including chromosome and molecular abnormalities are important diagnostic and prognostic factors in acute myeloid leukemia (AML). ATRA and imatinib have been successfully used in AML and chronic myelogenous leukemia, which proved that targeted therapy by identifying molecular lesions could improve leukemia outcomes. Recent advances in next generation sequencing have revealed molecular landscape of AML, presenting us with many molecular abnormalities. The individual prognostic information derived from a specific mutation could be modified by other molecular lesions. Therefore, the genomic complexity in AML poses a huge challenge to successful translation into more accurate risk stratification and targeted therapy. Herein, a summary of these mutations and targeted therapies are described. We focus on the prognostic information of recent identified molecular lesions and emerging targeted therapy.

## Background

**Fig. 1:**
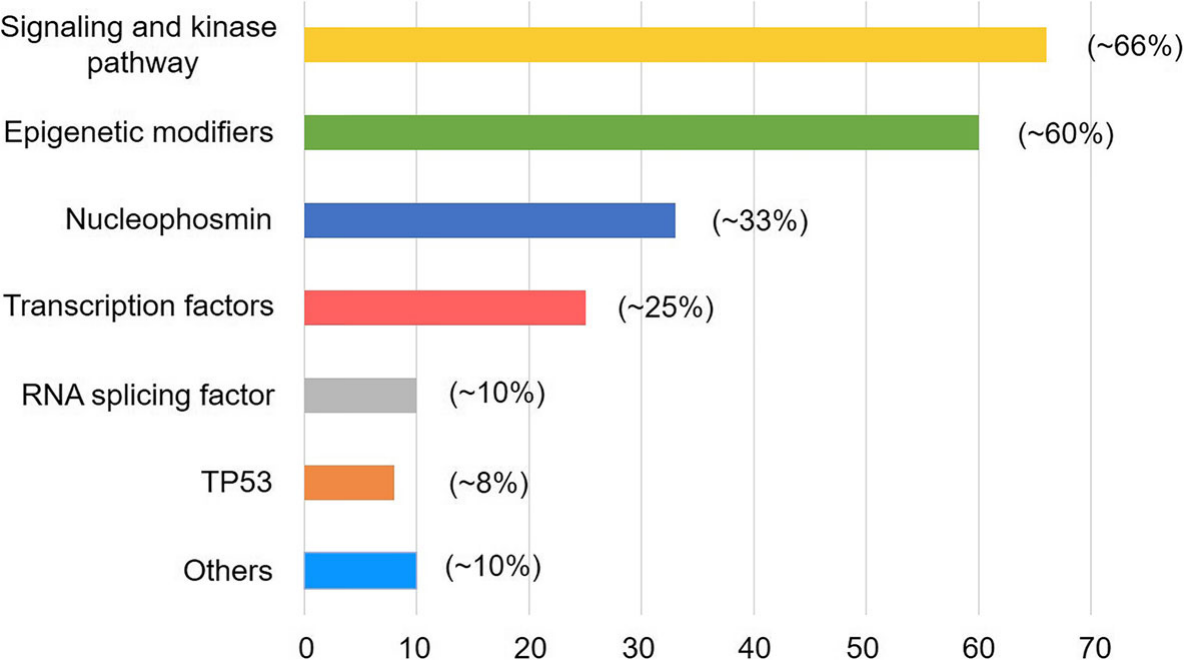
Distribution of recurrent AML mutations by functional group. A summary of the most frequent recurrent mutations in AML are listed in this figure. Other mutation as Cohesin complex are not discussed in detail in the manuscript. Mutational frequencies of each subgroup are derived from integration of data from several researches [1, 6, 8]

Acute myeloid leukemia (AML) is a heterogeneous disease, characterized by multiple somatically acquired driver mutations, coexisting competing clones, and disease evolution over time [1, 2]. Specific chromosomal aberrations and translocations have provided fundamental information in the evaluation of patients and guide for rational management. With advances in Next-generation sequencing (NGS) technologies, a detailed knowledge of the molecular landscape of AML has been discovered, with a better understanding in disease pathogenesis, classification and new therapeutic strategies [3–5]. More recently, German–Austrian AML Study Group revealed the cytogenetics and clinical data in 1540 patients with AML from three prospective trials (AML-HD98A, AML-HD98B and AMLSG-07-04). A total of 5234 AML driven mutations were identified and classified into non-overlapping 11 subtypes, which enabled us a better understanding of genomic landscape of AML from a macro perspective [1]. Encouraging efficacy of targeted therapy have brought about huge advance to AML treatment (details in Table 1) [6, 7]. A summary of these mutations and targeted therapy is described in the following sections (Fig. 1). Since fusion genes like PML-RARα, AML-ETO, CBFβ-MYH11 and MLL have been investigated for a long time, we wouldn’t discuss them here in our review.

**Table 1:** therapeutic targeting of individual AML mutations

Mutation	Therapeutic target	Inhibitors (phase of clinical trials)
FLT3	FLT3	FLT3 tyrosine kinase inhibitors: sorafenib (III), midostaurin (approved), quizartinib (III), crenolanib (III), gilteritinib (III), lestaurtinib (III) Other TKIs: ponatinib (I/II)
IDH1/2	IDH1	Ivosidenib (approved), IDH-305(I), BAY1436032(I), FT-2102(I/II), AG-881(I)
	IDH2	Enasidenib (approved), AG-881(I)
	BCL-2	venetoclax (III)
KIT	KIT	TKIs: imatinib, dasatinib (III), ponatinib
		sorafenib, sunitinib, quizartinib
TP53	TP53	PANDAS
	BCL-2	venetoclax
	MDM2	MDM2 inhibitors: RG7112 (I)
	Others	decitabine
SF3B1	SF3b complex	H3B-8800 (I)

*MDM2* mouse double minute 2 homolog, *SF3B1* splicing factor 3B subunit 1

### Nucleophosmin 1 (NPM1)

NPM1 mutations are reported in approximately one-third of AML adults, and more than half of them are with normal cytogenetics (CN-AML). It often co-occurs with mutations in epigenetic modifiers such as DNA methyltransferase 3A (DNMT3A), Ten-eleven translocation gene-2(TET2) and Isocitrate dehydrogenase1/2 (IDH1/2) mutations [6]. Numerous studies have confirmed that NPM1 mutations are an independent predictor of high CR rate and favorable prognosis in younger adults with AML, specifically in those without FMS-related tyrosine kinase 3-internal tandem duplications (FLT3-ITD) mutations [8, 9]. Recent studies have indicated that AML patients with NPM1 mutation and FLT3-ITD low allelic ratio may also have a more favorable prognosis regardless of chromosomal status, who should not be routinely assigned to allogeneic hematopoietic stem cell transplant (allo-HSCT) in the first complete remission [10, 11]. Patients harboring NPM1 mutations, even with high allelic ratio FLT3-ITD mutations have better prognosis than those with FLT3-ITD mutations alone [12]. In this setting, the latest ELN and NCCN risk stratification systems both classify NPM1 mutations with high allelic ratio FLT3-ITD mutations as intermediate risk group [13, 14]. However, the coexisting DNMT3A and FLT3-ITD mutations may predict the worst prognosis among AML patients with NPM1 mutation [1, 15]. It remains confirmed whether high NPM1-mutant allele burden at diagnosis predicts unfavorable outcomes in large prospective cohorts [16]. In elderly patients, NPM1 mutations are associated with a better CR rate, the prognosis of which has not been systematically confirmed. Most trials showed older CN-AML patients with NPM1 mutations have favorable treatment response and survival rate, while the prognosis of them is inferior to younger patients on the whole [17, 18]. However, several researches did not find favorable outcome of NPM1 mutation in older patients. Which may be related to different treatment regimens [19]. As for therapies, in the E1900 trial, patients with NPM1 mutant AML exposed to high dose daunorubicin (90 mg/m^2^) derived an increase in median overall survival (OS) compared with Standard dose daunorubicin (45 mg/m^2^) therapy (16.9 m vs 75.9 m) [20]. Besides, whether patients with NPM1 mutation will benefit from all-trans retinoic acid or arsenic acid treatment remains further discussion [21, 22].

### Signaling and kinase pathway mutations

In addition to mutations in NPM1, mutations leading to aberrant activation and proliferation of cellular signaling pathways, including FLT3, KRAS, NRAS, PTPN11, NF1, and KIT, are present in approximately two-thirds of AML cases.

#### FLT3

Mutations in FLT3 mostly involves internal tandem duplications within the juxta membrane region (FLT3-ITD) and point mutations in the tyrosine kinase domain (FLT3-TKD). Previous studies have confirmed that, FLT3-ITD mutations are associated with higher relapse rate and poorer overall survival, particularly with a high ratio of mutant allelic burden [23, 24]. In recent years, with great efforts in developing protein kinase inhibitors targeting FLT3 mutations the prognosis of patients with FLT3-ITD mutation has been significantly improved. Generally, the first generation of FLT3 inhibitors mainly include sorafenib, sunitinib, midostaurin and lestaurtinib. Because of having broad inhibiting targets besides FLT3-ITD mutation, it may improve the prognosis in AML without FLT3-ITD mutation. A phase II SORMAL clinical trial from Germany demonstrated that sorafenib could improve 3-year EFS in full set of primary AML (40% vs 22%, HR = 0.64, *n* = 267) [25]. A phase III international prospective RATIFY study confirmed that, addition of midostaurin to standard induction chemotherapy could significantly increase OS vs placebo among AML adults with FLT3 mutation (median OS of 74.7 m vs 25.6 m, HR = 0.78, *n* = 717) [26]. These results attribute a lot on the approval of midostaurin by FDA in newly diagnoses AML with FLT3-ITD mutation. According to the latest ELN and NCCN guideline for AML, midostaurin combining with chemotherapy was recommended as the first line treatment in adult FLT3 mutated AML [13, 14]. Second-generation small molecule FLT3 inhibitors such as quizartinib (AC-220), crenolanib (CP-868596), and gilteritinib (ASP-2215), have shown potent activity. Overall response for single agent FLT3 kinase inhibitor in treating FLT3-ITD mutated relapsed or refractory (R/R) AML is 40-50% in phase I and II clinical trials [27–29]. The efficacy of quizartinib was tested in patients with R/R FLT3-ITD mutated AML compared to salvage chemotherapy in a QuANTUM-R Trial. The median OS was 27 weeks and 20.4 weeks for patients treated with quizartinib and salvage chemotherapy, respectively [30]. It is the first phase III trial to demonstrate improved OS with FLT3 inhibitors in the R/R FLT3-ITD mutated AML setting. Also encouraging, interim study results of a phase I study of the combination of gilteritinib with induction chemotherapy reported an composite complete remission rate of 91.3% in newly diagnosed AML with FLT3-mutation (*n* = 23) [31]. Adding crenolanib to standard induction chemotherapy in patients with FLT3-mutated AML may be associated with low relapse rate when HSCT is routinely taken. In addition, for the patients did not undergo HSCT, only one (1/7) of them has relapsed, suggesting that standard chemotherapy plus crenolanib may also provide durable remissions without HSCT [32]. FLT3 inhibitors such as quizartinib and sorafenib target the inactive conformation of the kinase domain and only inhibit FLT3-ITD. Other inhibitors such as crenolanib, gilteritinib, and midostaurin target both the active and inactive conformation and show activity against both FLT3-ITD and TKD mutations [33]. Currently, several trials have been initiated to investigate the role of FLT3 inhibitors in maintenance therapy, eradiating MRD, and the combination regimen with chemotherapy (NCT02421939, NCT02752035, NCT02927262, NCT03070093).

#### Kit

The receptor tyrosine kinase KIT is frequently mutated in Core-binding factor AML (CBF-AML), which is defined by the occurrence of t (8;21) or inv.(16)/t (16;16) rearrangements. It has been demonstrated that the frequency of inv.(16)/t (16;16) AML in CBF leukemia is higher in Caucasian than in Chinese and Japanese [34]. The main mutational clusters in CBF-AML are commonly observed in KIT exon 8 and exon 17. It has been reported that mutations in KIT exon 17 or delayed reduction of RUNX1-RUNX1T1 transcripts conferred a higher risk of relapse and inferior OS in AML with t (8;21) [35]. Recently, several researches confirmed that t (8;21) AML patients with KIT mutations have a lower responsive rate after relapse. The prognostic impact of KIT mutations in inv.(16)/t (16;16) AML remains controversial [36]. Our recent study found that t (8;21) AML patients bearing KIT-D816 mutations have lower remission rates than those with wild-type KIT [37]. Patients with CBF-AML may benefit from high dose Cytarabine [38]. That is quite different from patients with NPM1 mutation who benefited from escalating dosage of anthracyclines, which have been mentioned above. The CALGB10801 trial indicated that adding multikinase inhibitors with activity against KIT mutations to chemotherapy, like dasatinib, could result in the similar 2 years DFS and OS in CBF-AML patients with kit mutation compared to those without, suggesting that dasatinib might improve the prognosis of in CBF-AML patients with KIT mutations [39]. Unfortunately, for patients with high-risk CBF-AML in CR1, dasatinib alone failed to prevent relapse due to molecular primary resistance or recurrence. Clinical trials about dasatinib undertaken by Shanghai Ruijin Hospital and our hospitial in China (ChiCRT-IPR-15006862 and NCT03560908) and other centers (NCT00850382) are ongoing.

### Mutations in transcription factors

Mutations in transcription factors occur in 20- 25% of patients with AML, including myeloid transcription factors, Runt-related transcription factor 1 (RUNX1) and CCAAT/enhancer binding protein α (CEBPA).

#### RUNX1

RUNX1 mutations were reported in 5% to 10% of AML, and more in patients with secondary AML evolving from myelodysplastic syndrome [40, 41]. The 2016 revised WHO AML classification system has added mutated RUNX1 as a provisional entity [42]. Studies have confirmed RUNX1 mutations an independent predictor of poorer prognosis [40, 41]. Concomitant mutations with ASXL1, SF3B1, SRSF2, PHF6 have been reported to have negative impact on OS, whereas patients with the genotype RUNX1mut/IDH2mut had better clinical outcome [40]. In addition, mutation burden and wild-type allele loss of RUNX1 as well as additional mutations also have impact on prognosis of adult RUNX1-mutated AML. Both wild-type loss and > 1 RUNX1mut showed adverse impact on prognosis compared with 1 RUNX1mut (OS 5 m vs 22 m, *P* = 0.002; 14 m vs 22 m, *P* = 00.048). Concomitant ASXL1 mutation and ≥ 2 additional mutations correlated with shorter OS (10 m vs 18 m, *P* = 0.028; 12 m vs 20 m, *P* = 0.017) [43].

#### CEBPA

Mutated CEBPA gene occur in 5-14% of AML patients, mainly in CN-AML, and was related to better prognosis. Recent studies showed that, the favorable impact of mutant CEBPA on prognosis is associated with biallelic mutations [44, 45]. The 2016 revision to the WHO classification system redefined the provisional WHO 2008 entity AML with CEBPA mutations to those with biallelic mutations [42]. Although the response rate for CEBPA double mutants is up to 90%, with estimated 5 years OS ranging between 50 and 70%, relapse still remains the major cause of treatment failure. The estimated cumulative incidence of relapse (CIR) of CEBPA dm patients after 5 years is up to 58% for intensive chemotherapy [46]. Richard, et al. analyzed 124 AML patients with CEBPA dm who achieved CR1. They found that the relapse-free survival (RFS) was significantly higher in patients receiving HSCT in CR1 compared with chemotherapy, whereas the OS was not different, which might be associated with a high second CR rate after salvage therapy [46].

### Mutations in epigenetic modifiers: Regulation of DNA methylation and chromatin modification

In recent years, the discovery of epigenetic regulators has provided great insight into the pathogenesis of AML. Mutations in these genes such as IDH1/2, DNMT3A, TET2, Additional sex comb-like 1(ASXL1), and Enhancer of zeste homolog 2(EZH2), which appear to impact on DNA methylation or histone posttranslational modifications, may serve as prognostic markers for risk stratification and therapeutic decision [47–49].

#### IDH1/2

Mutations in genes encoding IDH1 and IDH2 gene mainly involves IDH1-R132, IDH2-R140, and IDH2-R172, and IDH1 and IDH2 mutations rarely co-occur. IDH1-R132 or IDH2-R140 frequently occur in AML patients with normal cytogenetics and advanced age, with concurrent mutations of NPM1 [50]. While IDH2-R172 may represent a distinct genomic subgroup, which mutual exclusivity with NPM1 and with a distinct DNA methylation profile [51]. Experimental evidence demonstrates that IDH1/2 mutation occurs in the origin of clone, which is insufficient to induce leukemic transformation alone. Early data suggested that IDH mutations were associated with adverse prognosis, while recent results from a large cohort suggested that IDH mutations of different sites and additional co-occurring mutations may result in different outcomes [2, 51]. Several evidences showed that IDH1 and IDH2-R172 mutation may predict a worse clinical outcome especially in CN-AML, while the IDH2-R140 concomitant NPM1 mutation may be associated with better prognosis in AML [51–53]. Clearly, the prognostic impact of IDH1/2 mutations in AML has far been conflicting, and more efforts are needed for further study. Several small molecule inhibitors of IDH (AG-120, IDH-305 and FT-2102 for IDH1; AG-221 for IDH2; AG-881 for IDH1/2) have demonstrated potent preclinical activity, most of which are currently undergoing clinical trials and the early results are promising. The first-in-human phase I/II clinical trial of AG-221 (NCT01915498) for patients with IDH2-mutant advanced hematologic malignancies reported an objective response rate (ORR) of 40.3% (71/176) in R/R AML patients, with 19.3% CR rate and a median response duration of 5.8 months [54]. Interim study results of AG-120 reported an 41.9% ORR, including a 24.0% CR in IDH1-mutant R/R AML (*n* = 179) [55]. Based on its convincing therapeutic effects and great tolerance, ivosidenib (AG-120) and enasidenib (AG-221) had been approved by FDA for treatment in adult patients with R/R IDH1 and IDH2 mutant AML respectively. Studies investigating IDH inhibitors as monotherapy or combination regimen are still ongoing (NCT02632708, NCT02677922, NCT01915498, NCT02577406, NCT02719574, NCT03127 -735, NCT02492737). Moreover, patients with IDH mutations are found to be more responsive to B-cell CLL/lymphoma 2 (BCL-2) inhibitor [56].

#### DNMT3A, TET2 and ASXL1

Mutations in epigenetic regulators also include DNMT3A, TET2 and ASXL1, namely DTA mutations. These mutations are most common in people harboring age-related clonal hematopoiesis. Nowadays, no consensus has been reached on the prognosis of DNMT3a mutation. Some studies reported its poor prognosis [57], while others failed to find adverse impact [58]. Current researches suggested that DNMT3a mutation conferred adverse prognosis in AML patients with NPM1 mutation [59]. AML patients harboring NPM1, FLT3-ITD and DNMT3a mutations are associated with the worst prognosis [1]. Most recently, the latest ELN and NCCN risk stratification systems both classify ASXL1 mutations as adverse-risk AML subtypes. However, ASXL1 mutations should not be used as an adverse prognostic marker if they cooccur with favorable-risk AML subtypes [14, 60]. A recent series of studies demonstrated that, DNMT3A, occurred at a very early stage among genetic abnormalities, possessing a selective proliferative advantage which might be preleukemic events [61–63]. And in vivo experiments showed that mutated ASXL1 lowered the threshold of meningioma-1 driven engraftment, although it was insufficient to lead to blood malignancies [64]. Results from a systematic study involving 482 AML patients younger than 65 years old showed that DTA mutations remained detectable even during CR, and the persisting rates were 78.7% for DNMT3A, 54.2% for TET2, and 51.6% for ASXL1. The detection of persistent mutations did not correlate with an increased relapse rate with in a follow up period of 4 years (*P* = 0.29) [65, 66]. But a recent report revealed that the persistence of pre-leukemic mutation including DNMT3a might contribute to the inferior outcome of AML patients. Thus, the role of DNMT3a mutation during CR still needs further investigation [67].

### RNA splicing factor mutations

#### RNA splicing factor mutations

Mutations in splicing factors accounts for 10% of AML patients, which are defined by mutations in genes regulating RNA splicing (SF3B1, SRSF2, U2AF1 and ZRSR2). They are likely to cause aberrant splicing, affecting the transcriptome and proteome of cells. Accumulating evidence shows that spliceosome mutations are associated with older age, less proliferative disease, low response rate to standard treatment, and poorer survival [68]. Recent data suggested that many spliceosome inhibitors showed therapeutic potential in spliceosome mutant cancers. An orally available modulator of SF3b complex, H3B-8800, exhibits therapeutic potential of splicing modulation in spliceosome-mutant cancers in preclinical studies [69]. One phase I trial of H3B-8800 for patients with hematologic malignancies is currently ongoing (NCT02841540).

### Mutations in tumor suppressor genes

#### TP53

Although a lot of mutations are predicted to be activating or neomorphic, many of them have been demonstrated to be loss-of-function mutations, such as mutation in tumor protein 53(TP53), rendering them less tractable targets. TP53 is a key tumor suppressor gene. TP53 mutations accounts for 8% of patients with AML, and are associated with complex cytogenetics, therapy-related AML, chemoresistance, high relapse rates and poor survival [6, 58, 70]. Considering the fact that most of targeted drugs are gene inhibitors, as loss-of-function mutations, TP53 mutation is difficult to target. Although treat with intensive chemotherapy, the overall survival time is around 4.6 months [71]. It has been reported that decitabine, which decreases mutp53 levels, may improve the prognosis of patients with TP53 mutation, with a median survival of 12.7 months [72]. Besides, TP53 mutations have been described to be predictive for a favorable response to MDM2 inhibitors and BCL-2 inhibitors in hematologic malignancies [73–75]. Recently, Lu and his colleagues from Shanghai Jiaotong University identified a small molecule from thousands of compounds that can restore mutant TP53 with anti-cancer effect, which is named as PANDAS. Compared with previous TP53-targeting agents, PANDAS stabilizes molecular architecture of mutant TP53, restores its activity and promotes tumor cell apoptosis. The efficacy and tolerability of PANDAS, particularly in synergistic combinations, are keenly awaited.

## Conclusion and prospects

The discovery of the molecular landscape of AML not only provides us a chance to better understand the pathogenesis of the disease, but also refines the risk stratification and management of patients. However, evolving evidence demonstrated that the individual prognostic information derived from a specific mutation could be modified by other molecular characteristic and clinical parameters. Therefore, development of risk group classification schemes based on comprehensive genomic assessment should be considered a work in future. Targeted therapy to these mutations achieved huge progress in recent years. For instance, both IDH1/2 and FLT3 inhibitors showed promising results. And the combination of venetoclax with azacitidine produced a median OS longer than 12 months in older AML patients with a favorable safety profile [76, 77]. Considering the promising preliminary results of venetoclax, we can reasonably expect that the clinical outcomes for AML in older patients will be further improved in the foreseeable future.

In addition to genetic aberrations, epigenetics or posttranscriptional regulations may also play a pivotal role in determining the biological behavior of AML. Current researches demonstrated that DNA methylation patterns and long noncoding RNAs contribute to many critical signaling pathways in AML development, even exert effects on diagnosis classification and outcome of AML [78, 79]. And a further understanding of the relationship among genetic aberrations, DNA methylation, and long noncoding RNAs action might pave the way to better understand and treat AML patients.

In addition, encouraging efficacy of immunotherapeutic agents, especially the chimeric antigen receptor T (CAR-T) cell therapy, has brought huge advance to ALL treatment in the past decade [80–83]. However, previous trials of CAR-T therapy for AML did not result in long-term responses and exhibited the likelihood of hematopoietic toxicity, mainly due to the lack of AML-specific targeted antigens [84]. Currently, Liu and her colleagues reported on the robust anti-tumor activity and high safety of CAR-T cells targeting two different antigens simultaneously (CLL1-CD33 cCAR-T cells) [85]. This research unveiled a new strategy to circumvent unwanted off-target toxicity and contributed a significant step forward in AML immunotherapy. We believe that rational combination of targeted and immunotherapeutic agents will provide new insight into AML therapies and continue to accelerate progress in AML outcomes within the coming years.

## Abbreviations

allo-HSCT: Allogeneic hematopoietic stem cell transplant
AML: Acute myeloid leukemia
ASXL1: Additional sex comb-like 1
BCL-2: B-cell CLL/lymphoma 2
CAR-T: Chimeric antigen receptor T
CBF-AML: Core-binding factor AML
CEBPA: CCAAT/enhancer binding protein α
CIR: Cumulative incidence of relapse
CN-AML: Normal cytogenetics
DNMT3A: DNA methyltransferase 3A
EZH2: Enhancer of zeste homolog 2
FLT3-ITD: FMS-related tyrosine kinase 3-internal tandem duplications
FLT3-TKD: FMS-related tyrosine kinase 3-tyrosine kinase domain
IDH1/2: Isocitrate dehydrogenase1/2
MDM2: Mouse double minute 2 homolog
NGS: Next-generation sequencing
OS: Overall survival
R/R AML: Relapsed or refractory AML
RFS: Relapse-free survival
RUNX1: Runt-related transcription factor 1
SF3B1: Splicing factor 3B subunit 1
TET2: Ten-eleven translocation gene-2
TP53: Tumor protein 53
